# A Proteotranscriptomic-Based Computational Drug-Repositioning Method for Alzheimer’s Disease

**DOI:** 10.3389/fphar.2019.01653

**Published:** 2020-01-29

**Authors:** Soo Youn Lee, Min-Young Song, Dain Kim, Chaewon Park, Da Kyeong Park, Dong Geun Kim, Jong Shin Yoo, Young Hye Kim

**Affiliations:** ^1^ Research Center for Bioconvergence Analysis, Korea Basic Science Institute, Cheongju, South Korea; ^2^ Graduate School of Analytical Science and Technology, Chungnam National University, Daejeon, South Korea

**Keywords:** drug repositioning, Alzheimer disease, proteotranscriptomics, transcriptomics, proteomics, computational drug repositioning, drug discovery, system based approach

## Abstract

Numerous clinical trials of drug candidates for Alzheimer’s disease (AD) have failed, and computational drug repositioning approaches using omics data have been proposed as effective alternative approaches to the discovery of drug candidates. However, little multi-omics data is available for AD, due to limited availability of brain tissues. Even if omics data exist, systematic drug repurposing study for AD has suffered from lack of big data, insufficient clinical information, and difficulty in data integration on account of sample heterogeneity derived from poor diagnosis or shortage of qualified post-mortem tissue. In this study, we developed a proteotranscriptomic-based computational drug repositioning method named Drug Repositioning Perturbation Score/Class (DRPS/C) based on inverse associations between disease- and drug-induced gene and protein perturbation patterns, incorporating pharmacogenomic knowledge. We constructed a Drug-induced Gene Perturbation Signature Database (DGPSD) comprised of 61,019 gene signatures perturbed by 1,520 drugs from the Connectivity Map (CMap) and the L1000 CMap. Drugs were classified into three DRPCs (High, Intermediate, and Low) according to DRPSs that were calculated using drug- and disease-induced gene perturbation signatures from DGPSD and The Cancer Genome Atlas (TCGA), respectively. The DRPS/C method was evaluated using the area under the ROC curve, with a prescribed drug list from TCGA as the gold standard. Glioblastoma had the highest AUC. To predict anti-AD drugs, DRPS were calculated using DGPSD and AD-induced gene/protein perturbation signatures generated from RNA-seq, microarray and proteomic datasets in the Synapse database, and the drugs were classified into DRPCs. We predicted 31 potential anti-AD drug candidates commonly belonged to high DRPCs of transcriptomic and proteomic signatures. Of these, four drugs classified into the nervous system group of Anatomical Therapeutic Chemical (ATC) system are voltage-gated sodium channel blockers (bupivacaine, topiramate) and monamine oxidase inhibitors (selegiline, iproniazid), and their mechanism of action was inferred from a potential anti-AD drug perspective. Our approach suggests a shortcut to discover new efficacy of drugs for AD.

## Introduction

AD is the most common type of dementia, and is characterized by progressive declines in memory and cognition. The prevalence of AD is increasing rapidly as population ages. There are currently approximately 50 million people worldwide with dementia, and the cost of treating and caring for people with dementia is estimated to be about US$1 trillion per year (Patterson, 2018). Although the precise cause of AD is still unclear, the disease is characterized by the presence of amyloid plaques comprised of beta-amyloid (Aß) and neurofibrillary tangles (NFTs) comprised of hyperphosphorylated tau in the brain. Most drugs under development target these two pathological hallmarks. However, the success rate of newly-developed AD drugs has been very low, about 0.4%, and there have been hundreds of failures of clinical trials (Cummings and Science, 2018). Of all the potential drugs developed for the treatment of AD, only drugs such as cholinesterase inhibitors and memantine have been approved by the U.S. Food and Drug Administration (FDA) to relieve some of the symptoms of the disease. Given the impact of AD, it is therefore important to explore new drug development strategies for this condition.

Numerous drug repositioning methods have been suggested to repurpose already-approved drugs, and several compounds have been identified as innovative approaches to different diseases. Drugs that have been repositioned have undergone clinical trials, and so have confirmed pharmacokinetics, pharmacodynamics, and well-understood toxicity mechanisms, and have been approved by the U.S. FDA. Drug repositioning takes advantage of the reduced toxicity, side effects, and costs of clinical trials. Many computational drug repositioning methods based on transcriptomic data have been developed to identify potential new indications for drugs. Each method has applied techniques such as comparison of gene expression profiles between a disease model and the drug-treated condition (Chen et al., 2017), network integration (Luo et al., 2017), prediction of drug-protein interactions (Yang and Agarwal, 2011), and utilization of genotype-phenotype associations (Zhang et al., 2016). Systematic computational drug repositioning methods using large transcriptomic datasets perturbed by drugs have been developed (Dudley et al., 2011), and many promising drug candidates have been identified for diverse diseases (Végner et al., 2013; Zerbini et al., 2014). To assist in this endeavour, CMap (Lamb et al., 2006) and L1000 of the Integrated Network-based Cellular Signatures (LINCS) project (Subramanian et al., 2017) have been widely used. The CMap database was first released in 2006 and consisted of data relating to 564 gene expression signatures as perturbed by 164 bioactive small molecules. In 2010, the NIH LINCS consortium launched L1000, a database comprising approximately one million gene expression profiles of human cell lines as perturbed by about 15,000 drugs or small molecules. TCGA is the largest public data set related to human cancer genomes, and consists of multi-omics data generated by RNA-seq, copy-number variation analysis, genomic mutation, and DNA methylation, generated from 11,000 patients across 33 tumor types, together with relevant clinical information, including list of prescribed drugs (Nagaraj et al., 2018). Several studies developed and validated their methods based on anti-correlation between disease- and drug-induced gene expression profiles from these datasets. (Chen et al., 2017; Srivastava et al., 2018).

Most computational drug repositioning methods have been developed for a few diseases, such as cancers, since there are considerable amounts of gene and protein expression data available for these diseases, with clinical and pharmacological information, in databases such as TCGA. In the case of AD, little multi-omics data with clinical information have been produced, due to limitations in tissue availability from patients with clear clinical diagnoses. There have been several studies on the relationship between cancer and neurodegenerative diseases including AD, Parkinson’s disease (PD), and Huntington’s disease. Epidemiological studies have reported an inverse association between neurodegeneration and cancer, in that individuals with neurodegenerative diseases appear to have a lower risk of developing cancer and vice versa (Catalá-López et al., 2014; Seddighi et al., 2019). In addition, ageing-associated transcriptomic alterations are similar to those observed in neurodegeneration, but are opposite to those observed in cancer (Irizar et al., 2018). The expression of several genes that contribute to cell growth and proliferation is increased in cancer and decreased in AD (Shafi, 2016). There has, however, been some evidence of a positive association between AD and cancer. There appears to be a positive correlation between the mortality rates in AD and Glioma (Lehrer, 2018). This observation suggests a role of gene expression regulators in the shared genetic etiology between AD and cancer, and implies that some shared variants modulate disease risk. Increasing evidence suggest that there are common pathophysiological features in both diseases, such as DNA damage, oxidative stress, mitochondrial dysfunction, metabolic dysregulation, and inflammation (Driver, 2014; Houck et al., 2018). Moreover, single nucleotide polymorphism (SNP)-trait genome-wide association studies (GWAS) have shown positive genetic correlations between AD and cancer (Feng et al., 2017). Although the relationship between AD and cancer remains controversial, the analysis of large cancer multi-omics datasets and associated clinical information should provide insights into developing new drugs for AD.

In this study, we developed a new DRPS and new DRPC based on pharmacogenomic knowledge, along with the information that disease- and drug-induced gene and protein expression signatures have an inverse association. We first standardized drug names by PubChem compound identifier (CID) (Cheng et al., 2014b). Then we constructed a DGPSD comprised of 61,019 Drug-induced Gene Perturbation Signatures (DGPSs) generated by 1,520 compounds in 26 cell lines collected from CMap and L1000. DRPS was calculated using nine Cancer-induced Gene Perturbation Signatures (CGPSs) from 4,948 cancer and normal profiles (BRCA, UCEC, KIRC, LUAD, LUSC, COAD, STAD, CESC, and GBM) perturbed by 152 drugs, using data from TCGA, and each drug was classified into one of three DRPC (high, intermediate, low) by DRPS. The DRPS/C method was validated by calculating the AUC of each DRPC using DRPS as an input, and the prescribed drug list with CID as the gold standard. Glioblastoma (GBM) was found to have the highest AUC (0.708). Since GBM shared gene expression patterns and related pathways with AD, we applied the DRPS/C method to the prediction of anti-AD drugs using multi-omics datasets from AD patients. Two AD-induced Gene Perturbation Signature (AGPS) and one AD-induced Protein Perturbation Signature (APPS) were calculated from 159 RNA-seq, 108 microarray, and 17 proteomic datasets, respectively. We predicted 31 potential anti-AD drug candidates belonging to the intersection of high DRPCs that were calculated from AGPS and APPS. Of these, the mechanism of action of the drugs belonging to the nervous system class of ATC system was inferred from a potential anti-AD drug perspective. Our DRPS/C method may provide a shortcut to discover new efficacy of drugs for AD.

## Materials and Methods

### Standardization of Compound Names Based on PubChem Identifiers

When we investigated the collected compound lists that contained various nomenclature problems including uncertain naming, spelling errors, and the use of diverse synonymous. To solve these problems, we conducted cleaning and standardization of 1,858 compound names using CID of PubChem as follows. First, we selected 312 compounds that had the compound (“trt-cp”) or controls-vehicle (“ctl_vehicle”) perturbation type from LINCS level 3 data (GSE92742) (**Supplementary Table 3**). 159 and 1,387 compounds were extracted from the prescribed drug list of TCGA and CMap compounds list, respectively. Next, we converted compound names to CID using the PUG REST service provided by PubChem. For the un-mapped terms, we performed standardization of compound names into CIDs with human curation. Finally, we collected 1,608 compound names with CIDs (**Supplementary Figure 1**).

### Drug-Induced Gene Perturbation Signature Database (DGPSD)

Build02 (2009) of the CMap data was downloaded (https://portals.broadinstitute.org/cmap/index.jsp) and processed. We normalized the data with the MAS and quantile method using the affy R package (version 1.58.0). LINCS level 3 data were downloaded from the Gene Expression Omnibus (GSE92742). We selected 61,019 gene expression profiles from CMap (5,819) and L1000 (55,200), that have treated compounds with associated CID identifiers from our drug-CID mapping table (**Supplementary Figures 2A** and **3A**). In the case of the same experimental conditions (compound, treatment time, dosing, and cell line), we adopted the average of each gene expression value as a representative value of an experiment. To obtain DGPSs, the perturbed gene expression profiles induced by drugs, we calculated the log2 fold change in gene expression for each control versus compound-dosing-time experimental condition within the same cell line. To standardize the gene identifiers, all gene identifiers were converted to Ensembl gene IDs, using the BioMart R package (version 3.7).

### Analysis of Omics Expression Signatures

We downloaded prescribed drugs (40 types), patient information, prescribed drug list and normalized RNA-seq gene expression profiles from the TCGA (https://portal.gdc.cancer.gov, May, 2018). From these, we selected 4,948 gene expression data of nine cancer types (BRCA, GBM, CESC, COAD, KIRC, LUAD, UCEC, LUSC, STAD), which satisfied three conditions: (1) A cancer type dataset contained more than three normal samples with sample code 10 (solid tissue normal) or 13 (EBV immortalized normal); (2) the dataset included more than ten prescribed drugs; (3) It had one or more shared drugs from the drug lists from TCGA and CMap or L1000. In case of AD, 159 RNA-seq, 108 microarray, and 17 LC-MS/MS datasets were collected from Synapse (syn8690904), ArrayExpress (E-TABM-185), and PRIDE Archive (PXD006122, 4/6/2018), respectively (**Supplementary Data 4**).

The RNA-seq data were analyzed using Generalized linear models (GLM) in cancer versus unpaired normal samples (adjusted p-value < 0.05) using the R package EdgeR (release 3.7). All gene identifiers were transformed into Ensembl gene IDs using the BioMart R package (version 3.7) (**Supplementary Figures 2B**, **4A, B**, and **Supplementary Data 1**). Microarray data were normalized using the Robust Multi-array Average (RMA) algorithm and 817 differentially expressed genes were identified using t-test with a false discovery rate (FDR) correction (q-value < 0.05). The probe sets were summarized to Ensemble gene symbol using the HT HG-U133 database (version 3.7) and BioMart R package (version 3.7). The raw mass spectrometry data of AD human brain proteomics datasets were processed using Proteome Discoverer (Thermo Scientific, version 2.2) with a Uniprot human database (2017_08). Searches were performed using a 10 ppm precursor tolerance, and 0.05 Da fragment tolerance. Two missed cleavages were accepted. TMT tags on lysine residues and peptide N-termini (+ 229.162932 Da) and carbamidomethylation of cysteine (+ 57.02146 Da) were set as static modifications, while oxidation of methionine (+ 15.99492 Da) was set as a variable modification. Results were filtered to a 1% FDR at the peptide and protein levels. We normalized the quantitative proteome data using the VSN package (version 3.50.0) and performed t-test (p-value ≤ 0.05). Finally, we generated APPS based on 175 DEPs (**Supplementary Figures 4C, D**, and **Supplementary Data 2**). To comparison of biological characterization between AD and cancer, we performed pathway enrichment and PPI network analysis using GSEA v3.0 (MSigDB version 6.2, permutation method: 1,000 gene set) and STRING database (v10.5; confidence score of ≥ 0.8).

### Drug Repositioning Perturbation Score/Class

To calculate DRPS, we undertook the following analysis, First, for every gene *k* in each drug or disease gene expression profile perturbed by drug *j*, or disease *d*, we calculated log2 fold change (*F*_*drg*_*k*__; *F*_*dg*_*k*__) between drug-treated (*E_kj_*) and control (*E_kc_*); disease (*E_kd_*) and normal(*E_kn_*) gene expression profile, shown as Eq. 1 (Detail descriptions of the symbols in **Supplementary Table 1**).

(1) $$F_{drg_{k}}=\log_{2}\left(\frac{E_{kj}}{E_{kc}}\right),F_{dg_{k}}=\log_{2}\left(\frac{E_{kd}}{E_{kn}}\right)$$

Second, we identified differentially expressed genes in the intersection of DGPS and C/AGPSs Third, if gene *k* had an inverse signature expression pattern (*F*_*drg*_*k*__ > 0 & *F*_*dg*_*k*__ < 0, *F*_*drg*_*k*__ < 0 & *F*_*dg*_*k*__ > 0) between DGPS *j* and CGPS or AGPS *d*, we compute a Perturbation Score (PS) of gene *k* as follow Eq2.

(2) $$PS_{g_{k}}=\left|F_{dg_{k}}\right|-\left|F_{drg_{k}}\right|$$

To assign a weighted value to an influential pharmacogene using pharmacogenomic knowledge, we downloaded data from 11,922 pharmacogenes, including target, enzyme, transporter, and carrier from Drugbank (version 5.1.1; (Wishart et al., 2018) and defined these genes as our “PharmacoGene List” (PGL). We extracted pharmacogene (pg)s from DGPS *j*, or CGPS or AGPS *d* based on PGL (Eq.3) and computed log2 fold change (*F*_*drg*_*k*__/*F*_*dg*_*k*__) for every pg *i* as follow Eq.4. *E_ij_* and *E_ic_* is the gene expression value of pg *i* in drug-treated (*j*) and control(*c*) gene expression profiles. *E_id_* and *E_in_* are the gene expression value of pg *i* in disease(*d*) and normal(*n*) gene expression profiles.

(3) $$PG=\left\{g_{k}\in \mathrm{PGL}|pg_{1}\ldots pg_{i}\right\}$$

(4) $$F_{drpg_{i}}=\log_{2}\left(\frac{E_{ij}}{E_{ic}}\right),F_{dpg_{i}}=\log_{2}\left(\frac{E_{id}}{E_{in}}\right)$$

The PS of pg *i* was calculated in the same manner (*F*_*drpg*_*i*__ > 0 & *F*_*dpg*_*i*__ < 0, *F*_*drpg*_*i*__ < 0 & *F*_*dpg*_*i*__ > 0) as that of gene *k* (Eq.5).

(5) $$PS_{pg_{i}}=\left|F_{dpg_{i}}\right|-\left|F_{drpg_{i}}\right|$$

We calculated the DRPS of drug *j* (*DRPS*_*drug*_*j*__) as follows (Eq. 6). If drug *j* had multiple experimental conditions (dosing, time), we selected the maximum score among the DRPSs calculated from several experiment conditions (*e*). n and m are the total number of genes and pharmacogenes in the gene expression profile of DGPS *j* and CGPS or AGPS *d*.

(6) $${\mathrm{DRPS}}_{drug_{j}}=\overset{h}{\underset{e=1}{\max}}\left[\frac{1}{n}\sum_{k=1}^{n}PS_{g_{k}}\times \frac{1}{m}\sum_{i=1}^{m}PS_{pg_{i}}\right]$$

After sorted drugs by DRPS in ascending order, we classified into three DRPC (“high”, “intermediate”, “low”) based on DRPS.

## Results

### Compound Label Standardization and DGPSD Construction

**Figure 1:**
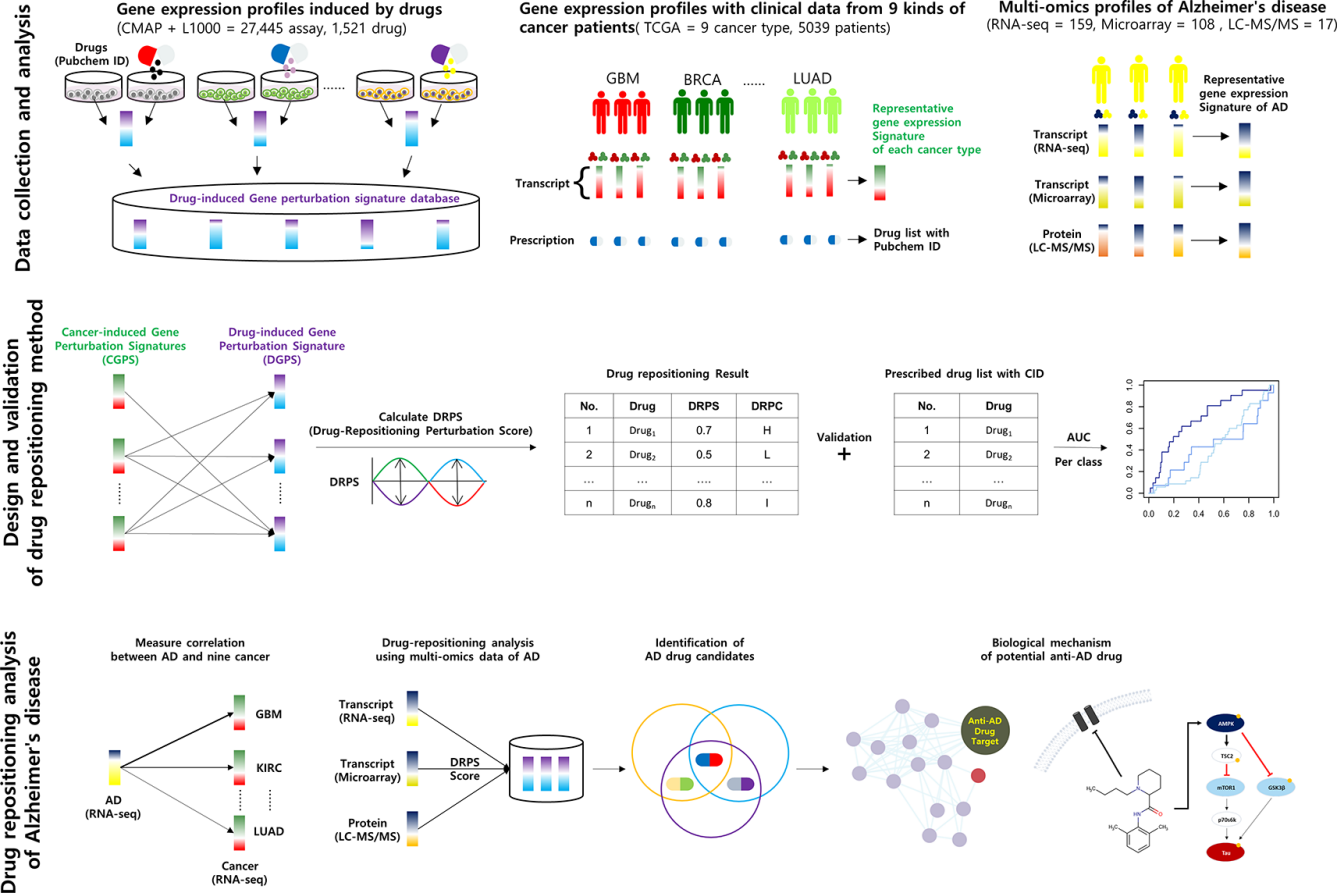
Schematic of the calculation of DRPS based on the inverse association between disease- and drug-induced transcript/protein perturbation signatures Higher DRPS means that the drug has not only a higher contrary correlation between drug-induced and cancer multi-omics signatures but also many influential pharmacogenes with high perturbation.

We collected 74,171 gene expression profiles perturbed by 2,021 compounds from CMap and L1000 to construct DGPSD. The DGPSD contained 15,137 and 14,123 genes from CMap and L1000, respectively. We standardized 1,858 compound labels from CMap (1,387), L1000 (312) and TCGA (159) based on the CID of PubChem, using the PUG REST service and human curation (**Supplementary Figure 1**). We selected gene expression profiles according to predefined criteria including drug name and CID. All gene expression profiles were converted into DGPS by calculating the log2-ratios of expression values between control and compound treated samples. The final DGPSD was made up 61,019 DGPS perturbed by 1,520 compounds in 26 cell lines (**Figure 1**; **Supplementary Figures 2A**, **3**).

### DRPS/C Method Development and Validation

**Figure 2:**
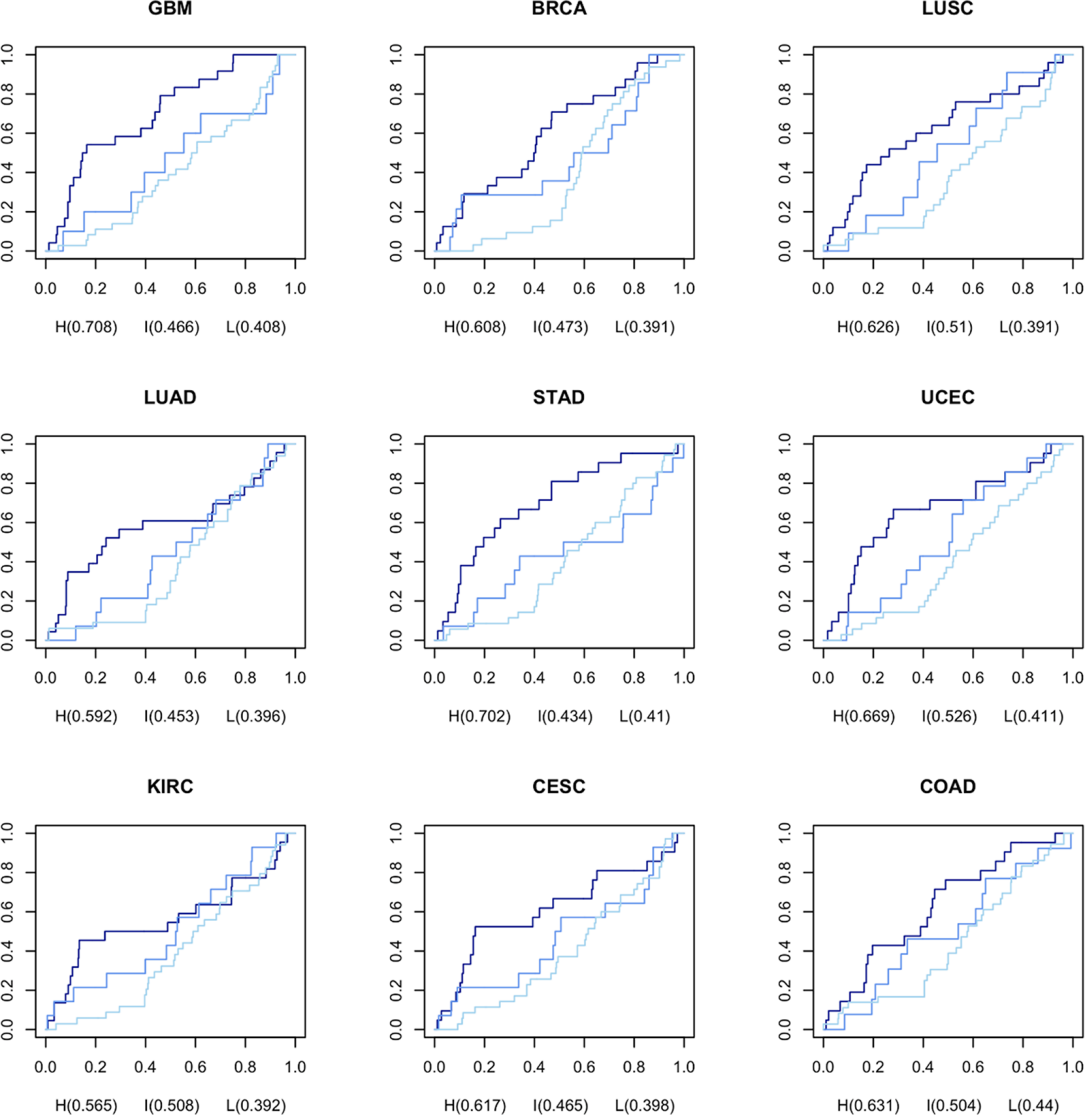
AUC for DRPS of each drug per DRPC using prescribed drugs as gold standard from TCGA The navy, medium blue, and light blue lines represent high, intermediate, and low classes in DRPC, respectively.

In order to generate CGPS, we selected gene expression profiles, meeting the criteria: the associated clinical data must include at least 20 kinds of prescribed drugs, and gene expression profiles should have one or more normal data sets. We computed nine CGPS through statistical analysis (Generalized linear models; adjusted P-value < 0.05; release 3.7) using 4,948 cancer and normal gene expression profiles perturbed by 152 drugs in nine cancer types (BRCA, UCEC, KIRC, LUAD, LUSC, COAD, STAD, CESC, and GBM) (**Supplementary Figure 2B**). Each CGPS included between 1221 and 4502 differential expressed genes (**Supplementary Data 1**, **Supplementary Table 2**, and **Supplementary Figure 4B**). We computed DRPS using DGPSD and nine CGPS. (**Supplementary Data 3**). The DRPS is a score that weights the pharmacogenomic knowledge supporting the value that measures an inverse association between each DGPS and CGPS.A higher DRPS means that the drug has not merely a higher inverse signature expression pattern between DGPS and CGPS, but also many influential pharmacogenes were perturbed. To select optimal drugs based on gene/protein expression data, we classified drugs based on DRPS and DRPC. To evaluate the performance of our method, we calculated the area under the ROC curve (AUC) of each DRPC for each cancer type using predicted repositioning candidate drugs ordered descending by DRPS score and prescribed drugs with CID from TCGA as a gold standard (**Figure 2**). The results show that the all AUCs in the nine cancer types were ordered as high, intermediate, and low class consistently. Based on these results, we assessed that the DRPS methods robustly predicts drugs based on inverse signature expression pattern. The highest AUC (0.708) was observed for GBM in the high class. We concluded that our DRPS is more valuable in drug repositioning analysis using brain gene expression data than when using data from other organs.

### Interrelation Between Cancer and AD

**Figure 3:**
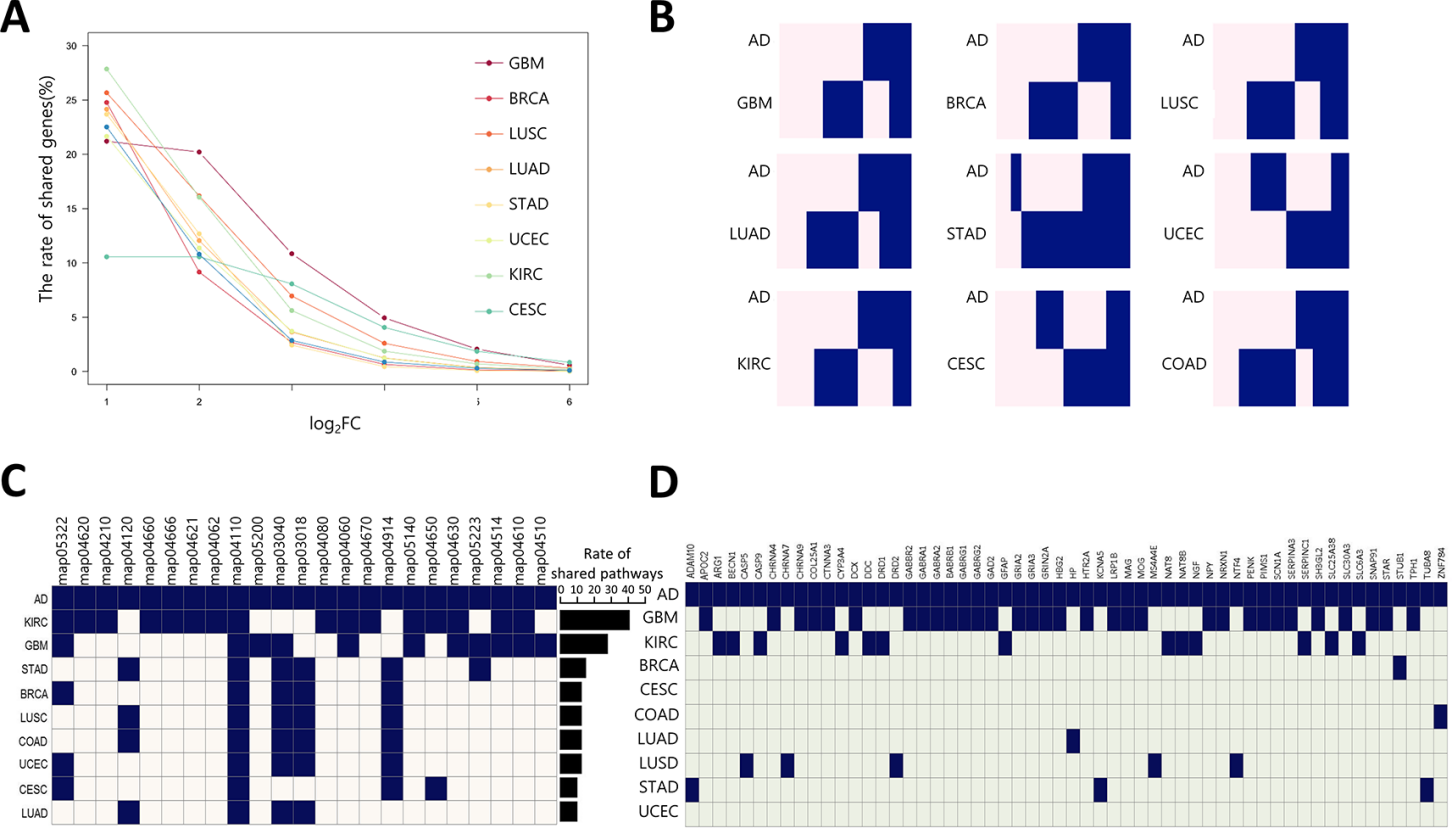
Correlation of gene expression between AD and cancer types **(A)** The ratio of shared DEGs between AD and nine cancer per each fold-change. **(B)** Gene-expression pattern similarity of AD and nine cancers. The pink and blue colors represent over-expressed and under-expressed DEGs, respectively. Coderivative of AD-related pathways **(C)** and genes **(D)** between AD and nine cancers. A navy square denotes an AD-related pathway in each cancer type, and light beige and light green indicates the opposite. The bar chart indicates rate of shared pathways between AD and each cancer type. KEGG pathway number description as follows: map05322, Systemic lupus erythematosus; map04620, Toll-like receptor signaling pathway; map04210, Apoptosis; map04120, Ubiquitin mediated proteolysis; map04660, T cell receptor signaling pathway; map04666, Fc gamma R-mediated phagocytosis; map04062, Chemokine signaling pathway; map04110, Cell cycle; map05200, Pathways in cancer; map03040, Spliceosome; map03018, RNA degradation; map04080, Neuroactive ligand-receptor interaction; map04060, Cytokine-cytokine receptor interaction; map04670, Leukocyte transendothelial migration; map04914, Progesterone-mediated oocyte maturation; map05140, Leishmaniasis; map04650, Natural killer cell mediated cytotoxicity; map04630, *JAK-STAT* signaling pathway; map05223, Non-small cell lung cancer; map04514, Cell adhesion molecules (CAMs); map04610, Complement and coagulation cascades; map04510, Focal adhesion.

To estimate the possibility of applying our method to AD drug repositioning, the relationship between cancer and AD was investigated at the transcription level. We downloaded 159 gene expression profiles generated by RNA-seq of AD brain tissues (AD: 88, Normal: 77) from the synapse portal, and computed AGPS comprised of 9,603 differentially expressed genes (DEGs). We measured the rate of shared DEGs between AGPS and CGPS per fold change (**Figure 3A**). GBM had the lowest reduction degree of shared DEG rates according to increasing fold change. These results indicate that AD and GBM share highly perturbed DEGs. We also calculated the rate of genes with same expression direction (overexpressed or underexpressed) in AGPS and CGPS. GBM (0.49) had highest similarity with AD. CESC (0.47) and STAD (0.46) followed (**Figure 3B**, **Supplementary Figure 5**). To assess whether AD and the nine cancer types share similar biological processes, we compared the significantly enriched pathways between AGPS and CGPS using the KEGG pathway gene sets in MSigDB (ver. 6.2) and GSEA v3.0. KIRC and GBM had the largest number of shared pathways with AD. *JAK-STAT* signaling pathway (map04630) and cytokine-cytokine receptor interaction (map04060) that were involved in long-term memory (Copf et al., 2011) were shared only in GBM and KIRC with AD (**Figure 3C**). In comparison of shared genes between CGPS with AD-related genes from Ingenuity Pathway Analysis (IPA) (Krämer et al., 2013), the GBM had the highest number of shared genes with AD (**Figure 3D**). PPI network analysis was also performed using the shared genes as an input for STRING, and the shared genes were linked with neurotransmitter receptors such as the glutamate, cholinergic receptor, and gamma-aminobutyric acid receptors (**Supplementary Figure 6**). Taken together, GBM showed a consistent strong correlation with AD.

### Drug Repositioning Analysis for AD Drug Discovery

**Figure 4:**
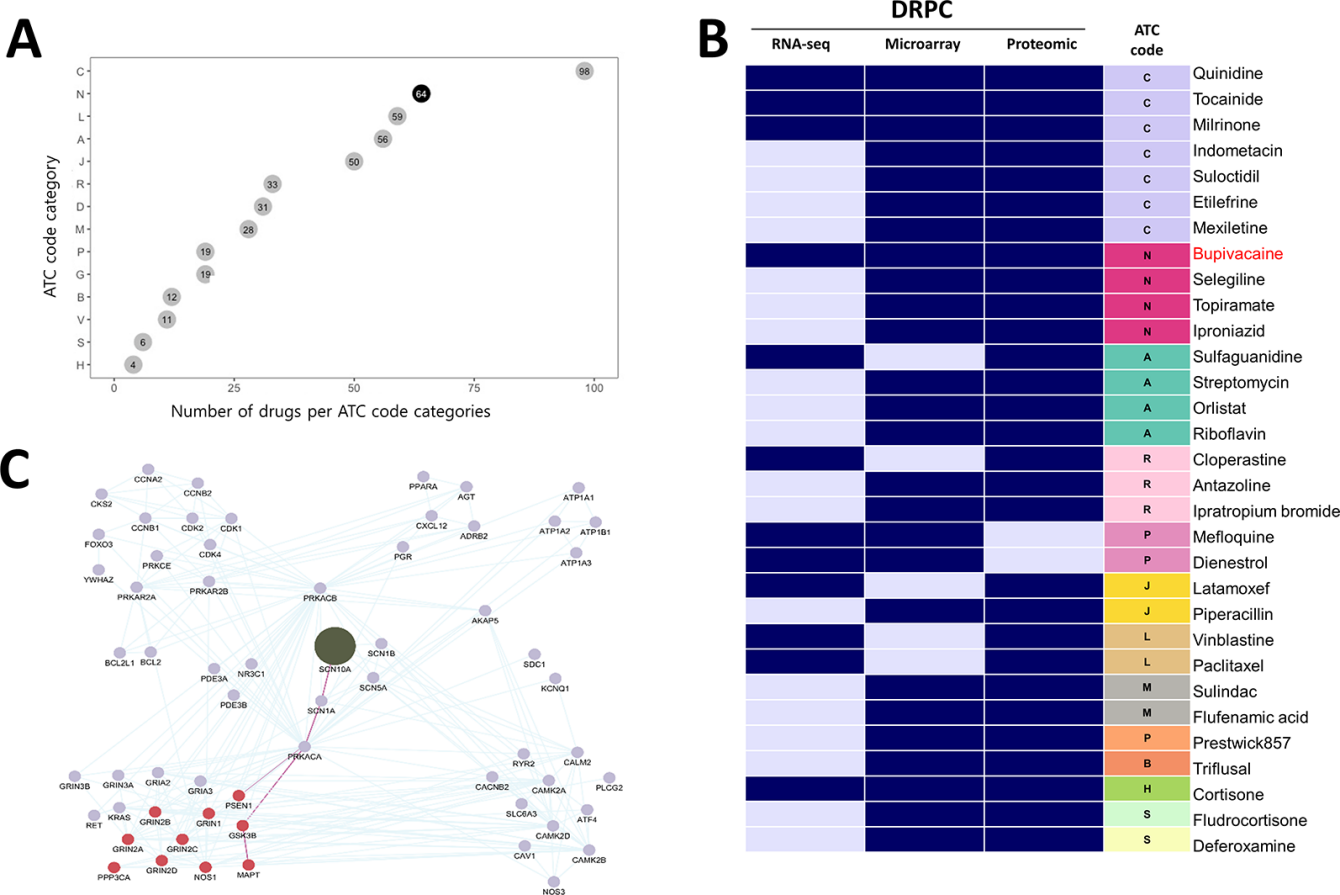
Potential anti-AD drugs with mode of action **(A)** The number of drugs per ATC code categories. **(B)** The DRPC per multi-omics data type with ATC class. Navy and sky-blue represent “high” and “intermediate” DRPCs, respectively. **(C)** The PPI network of *SCN10A* (olive node), the target protein of bupivacaine. Burgundy nodes denote AD-related proteins. The edges highlighted in purple denote the connectivity from SCN10A to PSEN1 or MAPT proteins, which are associated with AD pathological hallmarks.

To identify novel anti-AD drug candidates using DRPS/C, we further downloaded 108 and 17 gene expression profiles of brain tissues from AD patients generated by microarray, and LC-MS/MS from ArrayExpress database and PRIDE archive. The microarray data consisted of 22 AD and 86 normal samples, respectively. We computed microarray and RNA-seq AGPSs composed of 817 and 9,603 DEGs. Proteomic data included samples of 9 AD and 8 normal, and we generated AD-induced Protein APPS using 175 differentially expressed proteins (DEPs) (**Supplementary Data 2**). We then calculated the DRPS for each drug using 3 kind version of gene/protein expression signature AGPS (**Supplementary Data 4**). We found that 1,047 drugs were at least once ranked as high class (**Supplementary Data 3**). Among these drugs, 492 drugs had ATC code (ver. 2018). The most frequent drug class was C (Cardiovascular system, 98 drugs) and N (Nervous system, 64 drugs) (**Figure 4A**). We then selected 31 anti-AD drug candidates that were satisfied with the following criteria (**Supplementary Table 4**): 1) The drugs belonging to the intersection of high DRPCs from transcriptomic and proteomic data. 2) the drugs without low DRPC.

**Figure 5:**
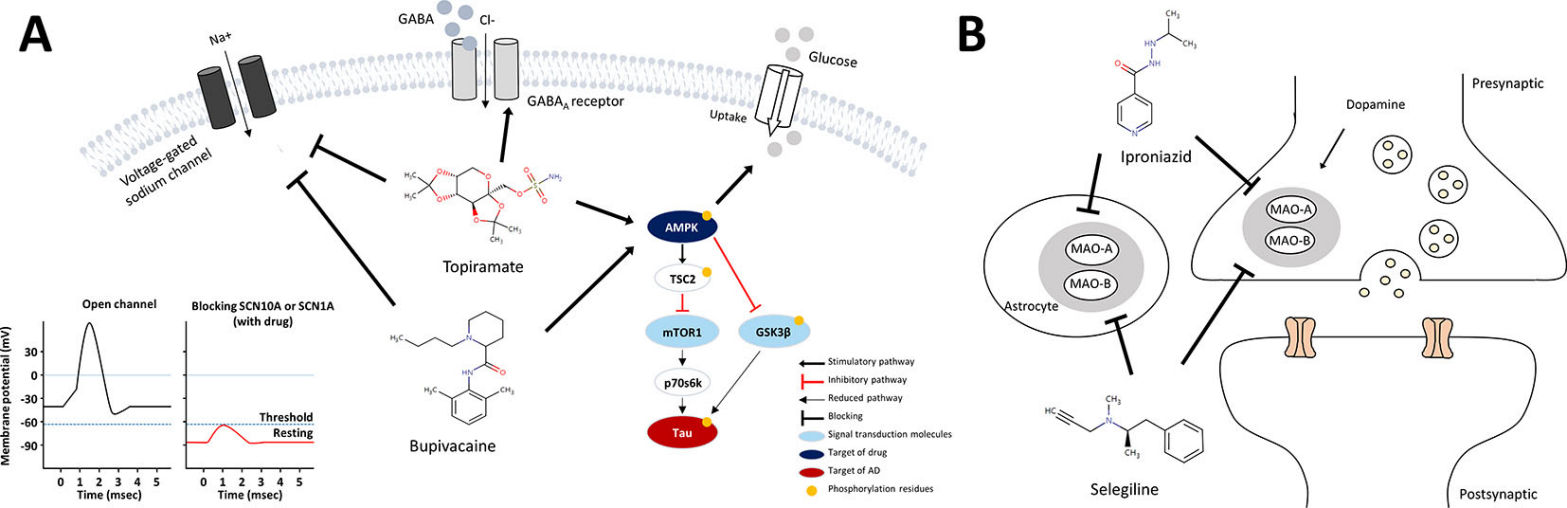
Schematic models for mechanism of action of anti-AD drug candidates in relation to AD pathology. **(A)** mechanism of action of the sodium channel blockers, bupivacaine and topiramate **(B)** mechanism of action of the MAO inhibitors, selegiline and iproniazid.

Of these, four drugs belonging to the ATC N class are bupivacaine, topiramate, selegiline and iproniazid (**Figure 4B**). We investigated the binding partners of bupivacaine target using AD-related genes from IPA and PPI relations from the STRING database. *SCN10A* (Sodium channel protein type 10 subunit alpha), a target of bupivacaine, was linked with *MAPT* (tau) and *PSEN1* (presenilin1), which were associated with the pathological hallmarks of AD, *via SCN1A* (sodium channel protein type 1 subunit alpha), a target of topiramate (**Figure 4C**). Bupivacaine and topiramate may inhibit neuronal hyper-excitability in AD by blocking sodium channel (Sheets et al., 2010) (**Figure 5A**). In another way, bupivacaine may act on AMP-activated protein kinase (AMPK), and subsequently activate the downstream of AMPK (Huang et al., 2014). Selegiline and iproniazid are inhibitors of monamine oxidase inhibitors (MAO) that are known to be implicated in the AD pathology (Thomas, 2000; Huang et al., 2012; Quartey et al., 2018) (**Figure 5B**). In this context, this approach can repurpose potential anti-AD drug candidates that may be further investigated.

## Discussion

Despite rapid increases in the prevalence of AD, therapeutic agents against AD have not yielded successful results in most clinical trials. Thus, treatment of AD urgently requires the development of novel, rationally designed therapeutic agents. Drug repositioning has attracted great interest, as it may lead to the discovery of novel drugs for diseases as well as reducing the risk of new drug development at the clinical stage. The commonly used computational drug repositioning methods started by searching for drugs that had an inverse association of gene expression pattern between disease and drugs. However, most approaches use transcriptomic data (Chen et al., 2017), and there have been few reports of a systematic drug repositioning method based on multi-omics data. Based on the inverse association, we developed a new method, DRPS/C, using public multi-omics data (transcriptomes and proteomes) incorporating pharmacogenomics knowledge. The DRPS/C method was successfully validated using a prescribed drug list in clinical data of cancer patients.

Since GBM outperforms the other cancer types by comparison of AUC values using the DRPS/C method, we further investigated gene expression pattern similarity, shared DEGs, and the related pathways between AD and GBM. GBM showed a consistent strong correlation with AD among nine cancer types. GBM is characterized by a high degree of cellular and molecular heterogeneity both among patients and within the same patient (Skaga et al., 2019). AD is also a heterogeneous disease that is classified into three clinical stages including the preclinical, mild cognitive impairment, and dementia (Jack et al., 2018), and its neuropathology is highly variable (Whitwell et al., 2012). For this reason, there might be far more complexities at the molecular level of these diseases. Advances in the diagnosis and single cell analysis as well as large scale multi-omics data for enough clinical samples may help investigation of the pathophysiological relationship between GBM and AD.

The most widely accepted theory to explain the pathogenic mechanism of AD is the amylod hypothesis, which states that the accumulation of Aß) leads to formation of amylod plaque and NFTs, ultimately, neuronal death (Selkoe and Hardy, 2016). Accumulating studies have shown various features in AD brain such as neuronal hyperexcitability, epileptic seizures, diminished glucose uptake, glutamate excitotoxicity, oxidative stress induced neurotoxicity, cholinergic hypofunction, metal dyshomeostasis, mitochondrial dysfunction, and neuroinflammation. Furthermore, these pathways are found to influence one another in the pathogenesis of AD (De Strooper and Karran, 2016).Although most current therapeutic approaches are focused on Aβ and hyperphosphophorylated tau, such complex features in AD have challenged the conventional paradigm in drug development. Among the anti-AD drug candidates predicted by using our methods, four drugs with the ATC nervous system code are voltage-gated sodium channel blockers (bupivacaine and topiramate) and MAO inhibitors (selegiline and iproniazid). According to the literature review, we inferred the mechanism of action of the drugs from a potential anti-AD drug perspective.

Bupivacaine, a FDA-approved local anesthetic, is known to block voltage-gated sodium channels by binding to SCN10A, inhibit *ionotropic glutamate receptors*, and activate AMPK (Lu et al., 2011). Topiramate, another sodium channel blocker to bind SCN1A, is approved to treat seizure disorders (Mantegazza et al., 2010). Topiramate has been known to modulate gamma-aminobutyric acid receptor subunit alpha-1(GABRA1) and glutamate receptors, and stimulate insulin-mediated glucose uptake by activation of AMPK (Caricilli et al., 2012). One of the characteristics of AD is neuronal hyper-excitability due to stimulated action potentials, which causes the loss of electrical signal transmission and ultimately neuronal death (Palop and Mucke, 2010). When increasing neuronal excitability, bupivacaine or topiramate may act on sodium channels to suppress neuronal action potentials (Sheets et al., 2010). Moreover, *SCN1A* connecting with *SCN10A* in the PPI network in **Figure 4C** is regulated by *BACE1*, the beta-site amyloid precursor protein cleaving enzyme for generation of Aβ peptides in AD. *PSEN1*, a component of γ-secretase producing Aβ, also mediates proteolytic cleavage of the voltage-gated sodium channel β-subunits (Kim et al., 2011). Glutamate receptor proteins, which are also related to neuronal excitability and affect synaptic plasticity *via JAK-STAT* signaling (**Figure 3C**), were indirectly linked with *SCN10A* and *SCN1A* (**Figure 4C**) (Nicolas et al., 2012). We thus inferred that bupivacaine or topiramate may prevent the neuronal cell damage in AD by regulating neuronal excitability.

In regards to AMPK activation, bupivacaine and topiramate might be associated with insulin-mediated glucose uptake (Caricilli et al., 2012) or tau phosphorylation through *AMPK*/*TSC2*/*mTOR1*/p70s6k pathway. Bupivacaine is known to activate *AMPK* along with T172 phosphorylation, and activated *AMPK* mediates the phosphorylation of S1387 in *TSC2* that initiates strong activation of the *AMPK*/*TSC2* pathway (Dibble et al., 2012; Huang et al., 2014). *mTOR1*, a central regulator of cell growth and metabolism, is inhibited by activated *AMPK*/*TSC2*. The mTOR-dependent *p70s6k* activity is also inhibited (Kickstein et al., 2010) and mediates *tau* phosphorylation, which is crucial in AD pathogenesis (Pei et al., 2006; Taga et al., 2011). Moreover, the activated *AMPK* inhibits activation of *GSK3β*, a major kinase of *tau* (Ryder et al., 2004; Horike et al., 2008). Combining all of these, we proposed the mechanism of action of bupivacaine and topiramate for the treatment of AD as shown in **Figure 5A**.

On the other hand, selegiline and iproniazid are inhibitors of MAO, a family of enzymes catalyzing the oxidation of monoamines. There are two types of MAO: MAO-A and MAO-B, and inhibition of MAO-A and MAO-B proteins increased dopamine in brain. Selegiline is used in the treatment of depression and early-stage Parkinson disease by modulation of dopaminergic transmission though blocking MAO-B (Finberg and Rabey, 2016). It is a selective irreversible MAO-B inhibitor in clinical doses, whereas it also inhibits MAO-A in larger doses (Fowler et al., 2015). Iproniazid, another MAO inhibitor, is used as an antidepressant drug (Yáñez et al., 2012). Several mechanisms have been proposed to account for involvement of MAO in AD pathology such as cognitive dysfunction *via* destroying cholinergic neurons and the formation of Aß aggregation or NFTs (Thomas, 2000; Huang et al., 2012; Mousseau and Baker, 2012; Cai, 2014; Quartey et al., 2018). This is in line with the recent study reporting that selegiline suppressed GABA production from reactive astrocytes, and restores the synaptic plasticity, and learning and memory function in the AD model mice (Park et al., 2019). Indeed, several studies showed beneficial effect of the MAO inhibitor, selegiline in AD (Tariot et al., 1987; Knoll et al., 1989; Sano et al., 1997), and dextroamphetamine, an inhibitor of MAO-A and MAO-B, is in Phase 4 clinical trial as a combination drug together with methylphenidate for AD treatment (Herrmann et al., 2008). Clinical trials have inherent limitations such that results can vary depending on patient population, dosage, duration of administration, and endpoint selection. Accordingly, there are still opportunities of applying these drugs for AD treatment.

However, for repositioning approved drugs in clinical trials, drug toxicity and unfavorable pharmacokinetics should be considered significant. Bupivacaine is primarily metabolized by the liver and should be used cautiously in patients with hepatic disease. There are also serious concerns about the systemic toxicity and cardiotoxicity of bupivacaine (El-Boghdadly et al., 2018). Topiramate is excreted predominantly in the urine as an unmetabolized drug and symptoms of overdose may cause vision problems, dehydration, metabolic acidosis, depression, encephalopathy, and kidney stones (Topiramate from Drugs.com, 2019). Selegiline is primarily metabolized by cytochrome P450 into L-desmethylselegiline, L-amphetamine, and L-methamphetamine in the liver and the intestines; they are excreted together with its metabolites in the urine and feces. However, amphetamine metabolites are also known to be associated with orthostatic hypotension and hallucinations (Romberg et al., 1995; Am et al., 2004). The side effects of selegiline include dizziness, insomnia, nausea, abdominal pain, skin rash, and weight loss (Selegiline from Drugs.com, 2019]. Iproniazid is a prominent mood stimulant for the treatment of debilitated individuals but was withdrawn from most markets because of its hepatotoxicity. The adverse effects of iproniazid also include dizziness, drowsiness, headaches, ataxia, numbness of the feet and hands, and muscular twitching (Lichtenstein and Mizenberg, 1954). Collectively, considering the pharmacokinetics and side effects of the repositioned drug candidates, further investigation of dose-dependent selectivity and interactions or development of specific drug moieties and targeted drug delivery systems must be undertaken.

We suggest expanding utilization of DRPS/C in diverse perspectives as follows. First, DRPS/C was designed for the easy addition of data from other biological signature including personal genomic variants by NGS, metabolomes, post-transcriptional translation, protein kinases, etc. If the other biological signatures are added, the anti-neuronal drugs predicted using our methods will be more reliable. Second, DRPS/C based on diverse biological signatures would be used in the strategic development of novel drug targets or biomarkers. Third, DRPS/C could be utilized in precision medicine. If we use a personal multi-omics expression profile instead of each disease multi-omics expression profile, our method will be able to suggest appropriate drugs for an individual.

In conclusion, DRPS/C method was developed to predict novel potential anti-neuronal drug candidates based on biological multi-omics signatures which reflected the inverse association and pharmacogenomics knowledge. Using the DRPS/C methods, we predicted potential anti-AD drug candidates including bupivacaine, topiramate selegiline, and iproniazid, and inferred their mechanism of action. Our approach suggests a shortcut to discover new drugs for AD. It may be also applicable to not only discovery of drug targets or biomarkers but also precision medicine.

## Data Availability Statement

Publicly available datasets were analyzed in this study. This data can be found here: https://www.ncbi.nlm.nih.gov/geo/query/acc.cgi?acc=GSE92742, https://portals.broadinstitute.org/cmap/, https://portal.gdc.cancer.gov/, https://www.synapse.org/#!Synapse:syn17010685, https://www.ebi.ac.uk/arrayexpress/experiments/E-TABM-185/, and https://www.ebi.ac.uk/pride/archive/projects/PXD006122.

## Author Contributions

SL, M-YS, and YK designed the study. SL developed the method algorithm. SL and M-YS performed omics data analyses. DK, CP, DP, and DGK participated in the construction of database, collection of dataset, and standardization of drug names. SL, M-YS, JY, and YK contributed to data interpretation and wrote the manuscript.

## Conflict of Interest

The authors declare that the research was conducted in the absence of any commercial or financial relationships that could be construed as a potential conflict of interest.

## Abbreviation

DRPS, Drug Repositioning Perturbation Score; DRPC, Drug Repositioning Perturbation Class; DGPSD, Drug-induced Gene Perturbation Signature Database; CGPS, Cancer-induced Gene Perturbation Signatures; DGPS, Drug-induced Gene Perturbation Signature; AGPS, AD-induced Gene Perturbation Signature; APPS, AD-induced Protein Perturbation Signature.
