# Supplementary material for: A Proteotranscriptomic-Based Computational Drug-Repositioning Method for Alzheimer’s Disease
Source: Front Pharmacol. 2020 Jan 29;10:1653. doi: 10.3389/fphar.2019.01653 (PMC7000455; doi:10.3389/fphar.2019.01653)
Supplement: Supplementary file 2 [file Presentation_1.pdf]

Supplementary Figure

A proteotranscriptomic-based computational drug-repositioning method for Alzheimer's disease

Soo Youn Lee[1#], Min-Young Song[1#], Dain Kim[1], Chaewon Park[1], Da Kyeong Park[1], Dong Geun Kim[1,2], Jong Shin Yoo[1,2*] and Young Hye Kim[1*]

[1] Research Center for Bioconvergence Analysis, Korea Basic Science Institute, Cheongju, Chungbuk, 28119, Republic of Korea

[2] Graduated School of Analytical Science and Technology, Chungnam National University, Daejeon, 34134, Re-public of Korea

[#] **Equal contribution**

*** Correspondence:**

Corresponding Author
E-mail: yhkim74@kbsi.re.kr (Kim YH)

Contents

Supplementary Figure 1. Workflow of selecting molecular signature expression profiles from CMap, L1000, and TCGA used to construct DGPSD.
Supplementary Figure 2. The results of compound name standardization. (A-B) Number of compounds with CID.
Supplementary Figure 3. Data composition of DGPSD.
Supplementary Figure 4. Data composition of cancer and AD gene expression profiles.
Supplementary Figure 5. The proportion of genes which had the same expression pattern in AD and nine cancer and named SEPG (Same Expressed Pattern Gene).
Supplementary Figure 6. The shared gene PPI network between AD and GBM.

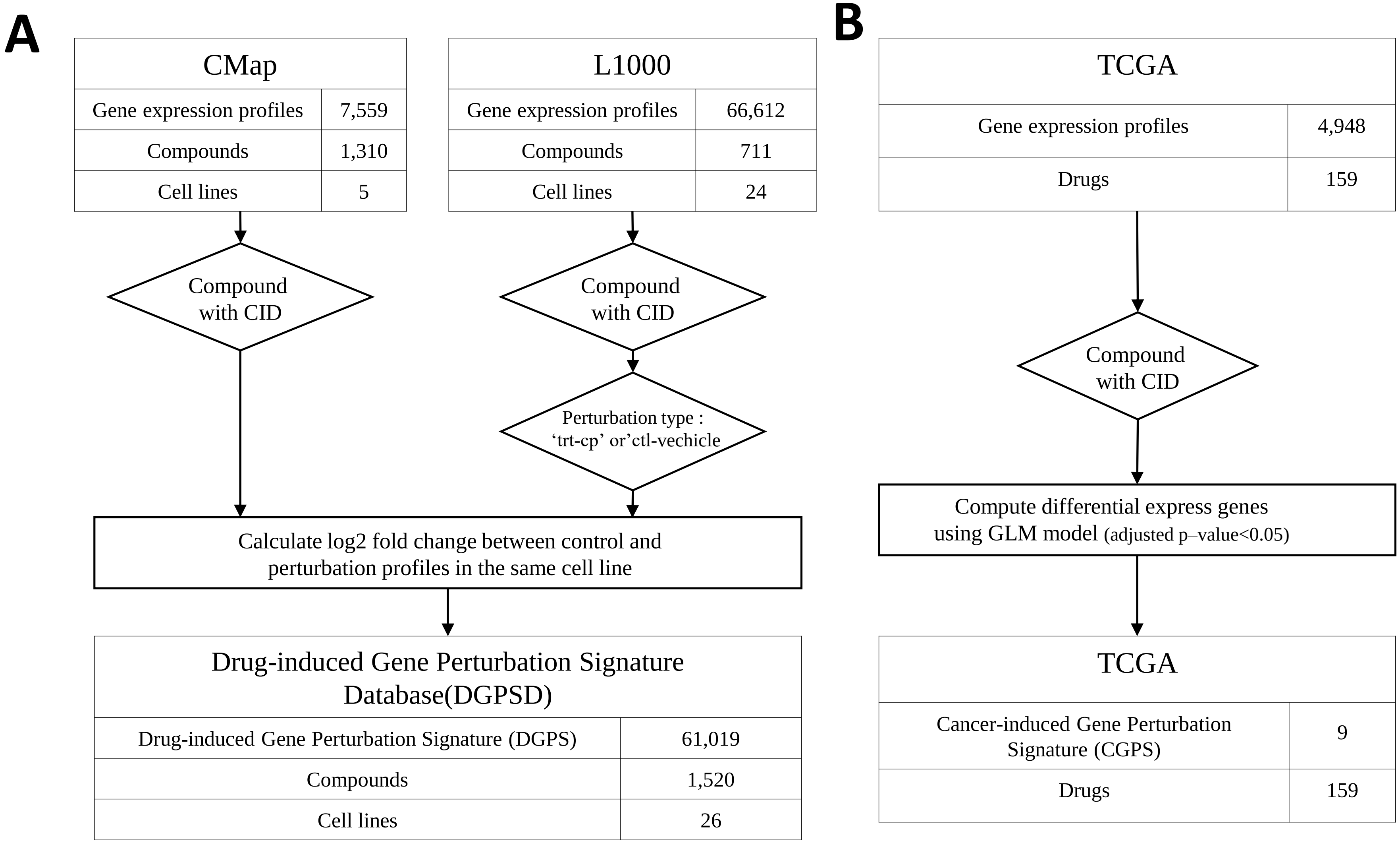

Supplementary Figure 1. Workflow of selecting molecular signature expression profiles from CMap, L1000, and TCGA used to construct DPGSD. The detailed method is described in the Methods section.

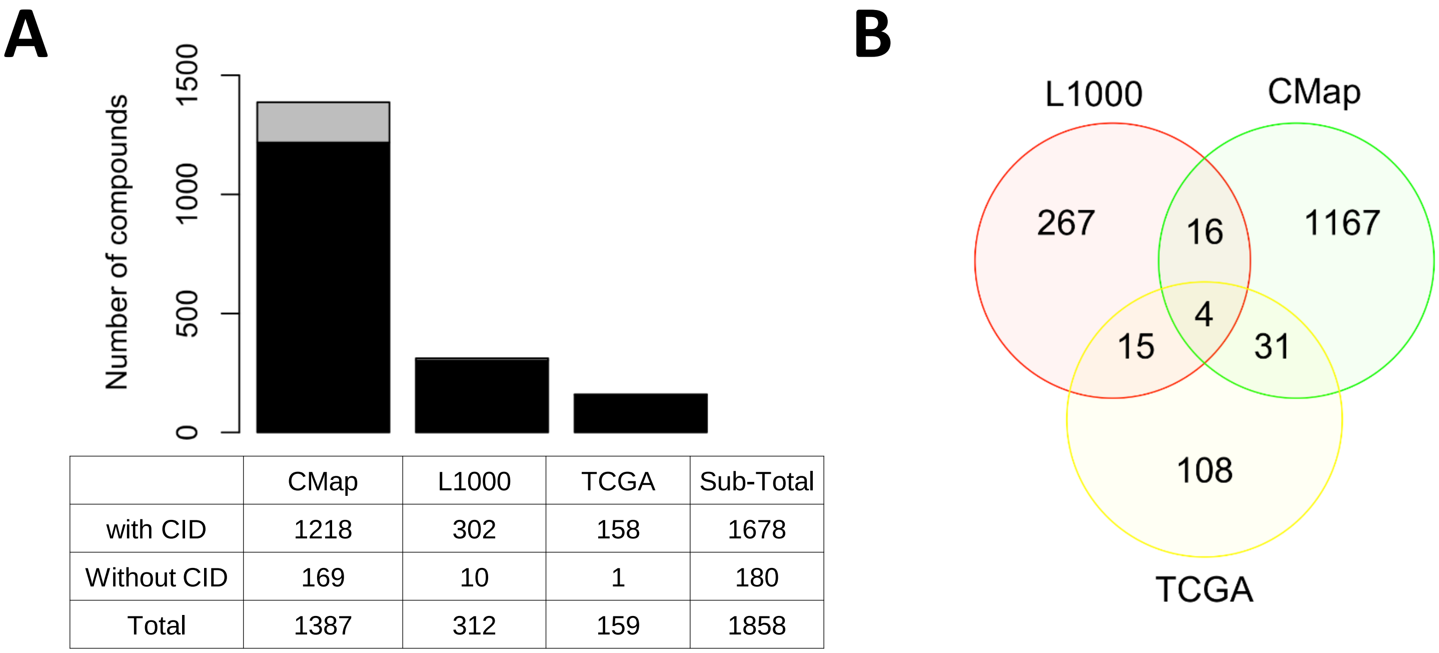

	CMap	L1000	TCGA	Sub-Total
with CID	1218	302	158	1678
Without CID	169	10	1	180
Total	1387	312	159	1858

Supplementary Figure 2. The results of compound name standardization. **(A-B)** Number of compounds with CID.

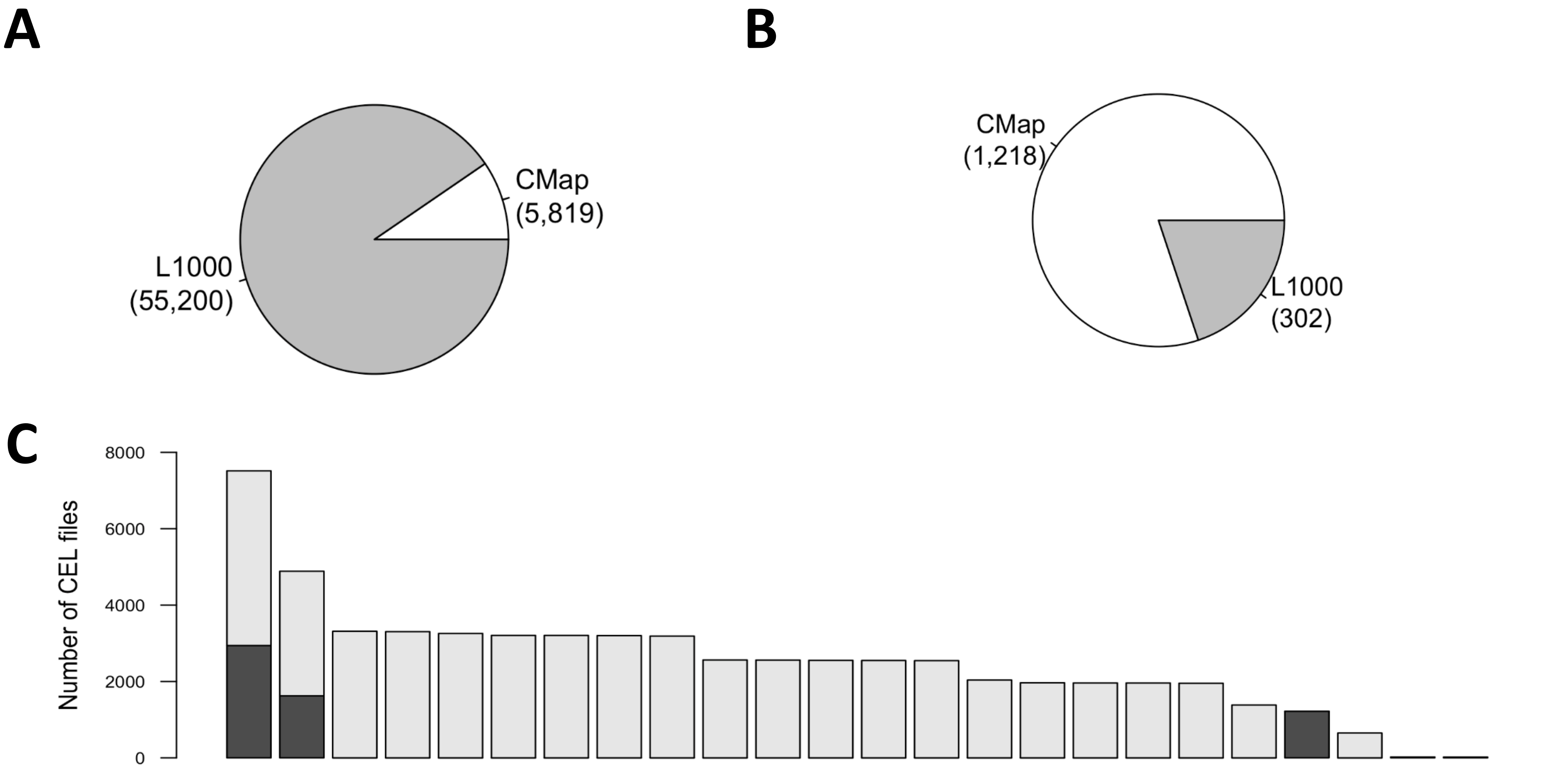

Cell line	MCF7	PC3	A375	HME1	A549	HCC515	HT29	HEPG2	HA1E	BT20	MDAMB231	SKBR3	HS578T	MCF10A	NPC.TAK	ASC.C	HUVEC	SKL.C	DC34	LNCAP	HL60	JURKAT	ssMCF7	SKMEL5	Total
CMap	2941	1624	0	0	0	0	0	0	0	0	0	0	0	0	0	0	0	0	0	0	1219	0	19	16	5819
L1000	4574	3264	3314	3306	3256	3207	3207	3203	3188	2564	2561	2551	2548	2545	2039	1968	1961	1960	1952	1383	0	649	0	0	55200
Total	7515	4888	3314	3306	3256	3207	3207	3203	3188	2564	2561	2551	2548	2545	2039	1968	1961	1960	1952	1383	1219	649	19	16	61019

Supplementary Figure 3. Data composition of DGPSD. Number of CEL files**(A)**, Compounds**(B)**, CEL files per each cell line**(C)** in DGPSD.

A

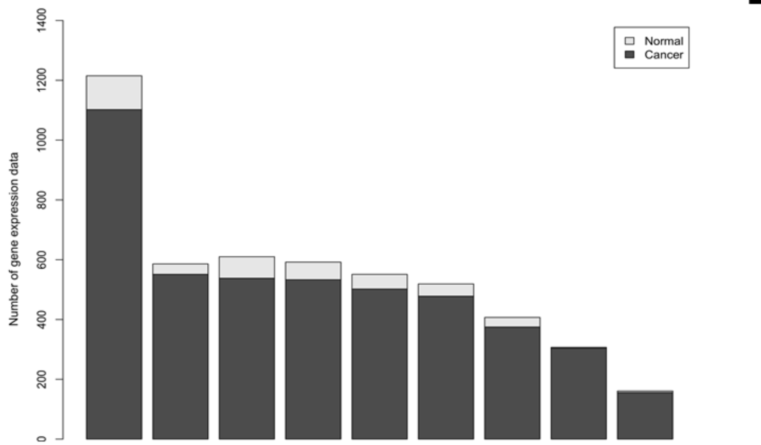

	BRCA	UCEC	KIRC	LUAD	LUSC	COAD	STAD	CESC	GBM	Total
#Caner data	1102	551	538	533	502	478	375	304	156	4539
#Normal data	113	35	72	59	49	41	32	3	5	409
Total	1215	586	610	592	551	519	407	307	161	4948

B

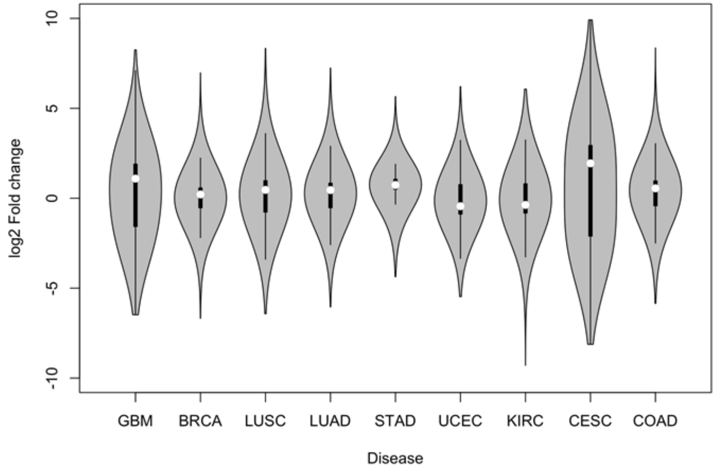

C

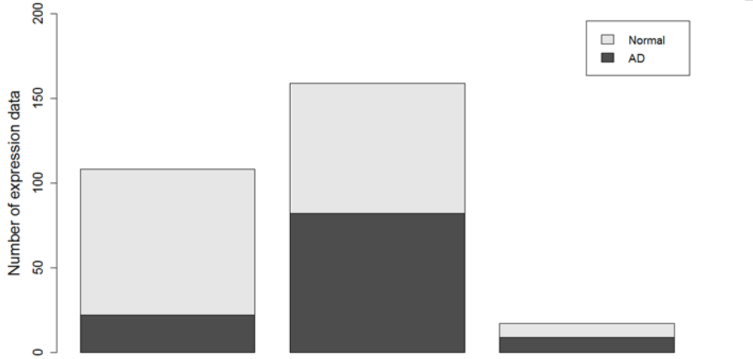

	Microarray	RNA-seq	Proteomic	Total
#Normal data	22	77	8	107
#AD data	86	82	9	177
Total	108	159	17	284

D

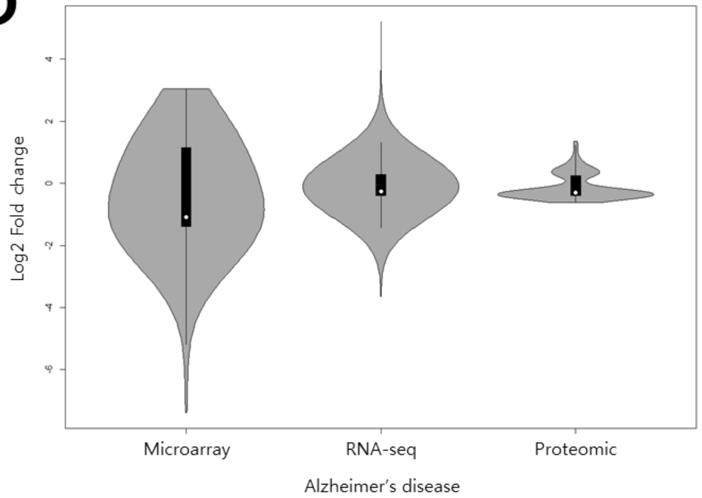

Supplementary Figure 4. Data composition of cancer and AD gene expression profiles. **(A)** Number of gene expression profiles for nine cancers from TCGA. **(B)** Log2 fold change distribution per cancer. **(C)** Number of molecular signature expression profiles per multi-omic data type. **(D)** Log2 fold change distribution per multi-omic data type.

A

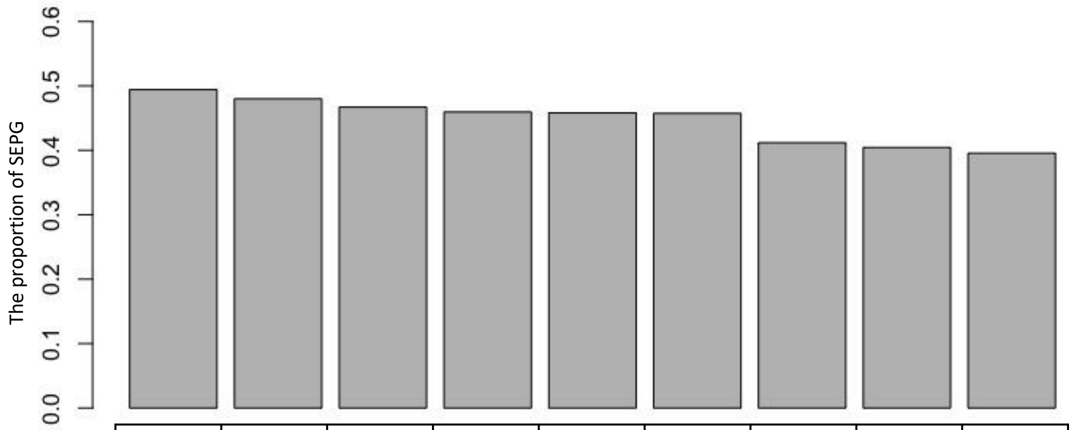

GBM	BRCA	LUSC	LUAD	STAD	UCEC	KIRC	CESC	COAD
0.49	0.39	0.45	0.45	0.46	0.40	0.41	0.47	0.45

Supplementary Figure 5. The proportion of genes which had the same expression pattern in AD and nine cancer and named SEPG (Same Expressed Pattern Gene).

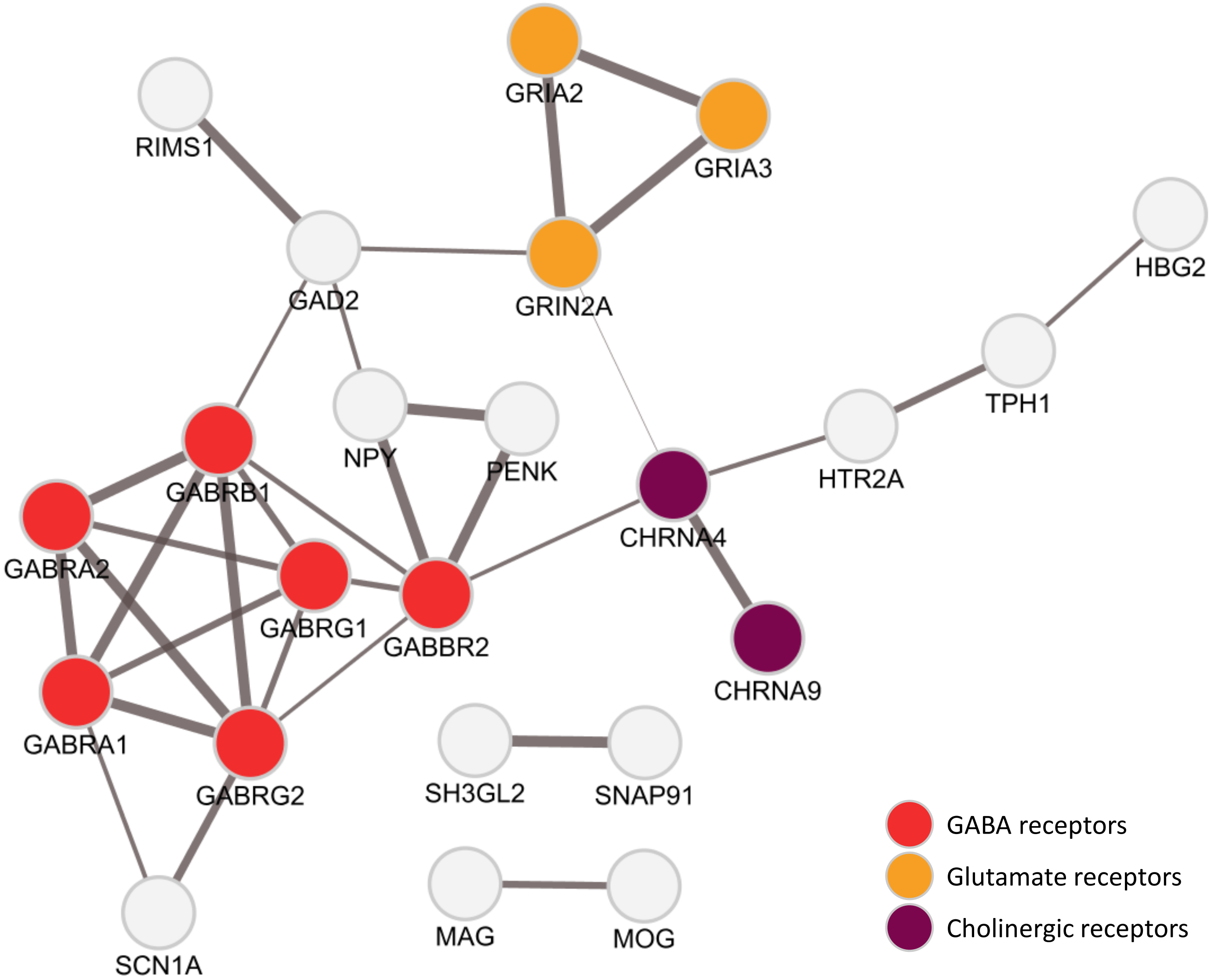

Supplementary Figure 6. The shared gene PPI network between AD and GBM. The degree of thickness denotes confidence score range (0.4 to 0.9). A thicker line indicates a higher confidence score (0.9). The red, orange and purple circles denote GABA, Glutamate, and Cholinergic receptor family proteins, respectively.
