# Supplementary material for: A Proteotranscriptomic-Based Computational Drug-Repositioning Method for Alzheimer’s Disease
Source: Front Pharmacol. 2020 Jan 29;10:1653. doi: 10.3389/fphar.2019.01653 (PMC7000455; doi:10.3389/fphar.2019.01653)
Supplement: Supplementary file 3 [file Presentation_2.pdf]

Supplementary Table

A proteotranscriptomic computational drug-repositioning method for Alzheimer's disease

Soo Youn Lee[1#], Min-Young Song[1#], Dain Kim[1], Chaewon Park[1], Da Kyeong Park[1], Dong Geun Kim[1,2], Jong Shin Yoo[1,2*] and Young Hye Kim[1*]

[1] Biomedical Omics Group, Korea Basic Science Institute, Cheongju, Chungbuk, 28119, Republic of Korea

[2] Graduated School of Analytical Science and Technology, Chungnam National University, Daejeon, 34134, Re-public of Korea

[#] **Equal contribution**

*** Correspondence:**

Corresponding Author
E-mail: yhkim74@kbsi.re.kr (Kim YH), jongshin@kbsi.re.kr (Yoo JS).

Contents

Supplementary Table 1. Descriptions for symbols of the DRPS/C method.

Supplementary Table 2. Number of differential expressed genes per log2fold and nine cancer types.

Supplementary Table 3. Data description.

Supplementary Table 4. Anti-Alzheimer’s disease drug candidates which were discovered by the DRPS/C method

Supplementary Table 1. Descriptions for symbols of the DRPS/C method.

Symbol	Description
PG	Pharmacogene set.
PGL	Pharmacogene list from DrugBank.
g_k	k^{th} gene
E_{kc}	mRNA expression value of kth gene in c^{th} control sample.
E_{kj}	mRNA expression value of kth gene in j^{th} drug treated sample.
E_{kn}	mRNA expression value of kth gene in n^{th} normal sample.
E_{kd}	mRNA expression value of kth gene in d^{h} disease sample.
E_{ic}	mRNA expression value of ith pharmacogene (*pg*) in c^{th} control sample.
E_{ij}	mRNA expression value of ith pharmacogene (*pg*) in j^{th} drug treated sample.
E_{in}	mRNA expression value of ith pharmacogene (*pg*) in n^{th} normal sample.
E_{id}	mRNA expression value of ith pharmacogene (*pg*) in d^{h} disease sample.
F_{drg_k}	Log_2 fold change value of kth gene between drug treated and control expression value.
F_{dg_k}	Log_2 fold change value of kth gene between disease and normal expression value.
F_{drpg_i}	Log_2 fold change value of ith pharmacogene (*pg*) between drug treated and control expression value.
F_{dpg_i}	Log_2 fold change value of ith pharmacogene (*pg*) between disease and normal expression value.
PS_{g_k}	Perturbation score of kth gene.
PS_{pg_i}	Perturbation score of ith pharmacogene.
$DRPS_{drug_j}$	Drug repositioning perturbation score of drug *j*

Supplementary Table 2. Number of differential expressed genes per log2 fold and nine cancer types.

Log2 FC	GBM	BRCA	LUSC	LUAD	STAD	UCEC	KIRC	CESC	COAD
0	3047	4103	4502	3982	4038	3197	4896	1221	3601
1	2850	1018	2100	1416	1546	1312	2020	1221	1269
2	1259	268	749	376	244	380	588	897	290
3	521	64	263	123	44	120	186	422	86
4	207	15	91	36	8	32	69	187	29
5	57	7	31	13	2	6	22	83	10
6	12	2	6	5	0	0	3	27	1
7	3	0	2	0	0	0	1	8	1
8	0	0	1	0	0	0	1	1	0
9	0	0	0	0	0	0	0	0	0

Supplementary Table 3. Data Description.

Data name	Data type	Data ID	Experiment technology type	No.Experiment data	Site
L1000	Drug-induced transcripomic Signature	GSE92742	Microarray (GPL20573)	7,519	https://www.ncbi.nlm.nih.gov/geo/query/acc.cgi?acc=GSE92742
cMAP	Drug-induced transcripomic Signature		Microarray	66,612	https://portals.broadinstitute.org/cmap/
TCGA	transcripomic Signature of personal cancer patients		RNA-seq	4,948	https://portal.gdc.cancer.gov/
	Clinical information of personal cancer patients				https://portal.gdc.cancer.gov/
Synapse	transcripomic Signature of AD patients	syn8690904	RNA-seq	159	https://www.synapse.org/#!Synapse:syn8690904
ArrayExpress	transcripomic Signature of AD patients	E-TABM-185	Microarray (HG-U133A)	108	https://www.ebi.ac.uk/arrayexpress/experiments/E-TABM-185/
PRIDE	Proteomic Signature of AD patients	PXD006122	LC-MS/MS	17	https://www.ebi.ac.uk/pride/archive/projects/PXD006122

Supplementary Table 4. Anti-Alzheimer's disease drug candidates which were discovered by the DRPS/C method

No.	Drug name	Pubchem CID	indication	ATC code	Target
1	Quinidine	441074	ventricular pre-excitation<br>cardiac dysrhythmias	C01BA01,<br>C01BA51,<br>C01BA71	Sodium channel protein type 5 subunit alpha,<br>UPotassium channel subfamily K member 1,6,<br>UPotassium voltage-gated channel subfamily H member 2,<br>UAlpha-1A,B,D adrenergic receptor
2	Tocainide	38945	ventricular arrhythmias	C01BB03	Sodium channel protein type 5 subunit alpha,<br>cGMP-inhibited 3',5'-cyclic phosphodiesterase A,<br>Prostaglandin G/H synthase 1, 2 and reductase 2
3	Milrinone	4197	congestive heart failure	C01CE02	cGMP-inhibited 3',5'-cyclic phosphodiesterase A
4	Indometacin	3715	rheumatoid arthritis<br>ductus arteriosus	S01CC02,<br>M01AB51,<br>S01BC01,<br>M02AA23,<br>M01AB01,<br>C01EB03	Prostaglandin G/H synthase 1, 2 and reductase 2,<br>Phospholipase A2, membrane associated,<br>Peroxisome proliferator-activated receptorn alphal, gamma,<br>Lactoylglutathione lyase,<br>Prostaglandin D2 receptor 2
5	Suloctidil	657255	vascular antispastic	C04AX19	-
6	Etilefrine	3306	cardiac stimulant<br>antihypotensiv<br>orthostatic hypotension of neurological, cardiovascular, endocrine or metabolic orig<br>in	C01CA51,<br>C01CA01	-
7	Mexiletine	4178	ventricular tachycardia, beats, fibrillation	C01BB02	Sodium channel protein type 5 subunit alpha,<br>Aryl hydrocarbon receptor
8	Bupivacaine	2474	local or regional anesthesia<br>obstetrical procedures	N01BB01,<br>N01BB51	Sodium channel protein type 10 subunit alpha,<br>Prostaglandin E2 receptor EP1 subtype
9	Selegiline	26757	initial treatment of Parkinson's disease<br>palliative treatment of mild to moderate Alzheimer's disease<br>depression	N04BD01	Amine oxidase [flavin-containing] A, B
10	Topiramate	5284627	tonic-clonic seizures<br>seizures associated with Lennox-Gastaut syndrom<br>migraine headach<br>weight reduction in patients with obesity or diabetes	N03AX11	Gamma-aminobutyric acid receptor subunit alpha-1,<br>GABA-A receptor (anion channel) (Protein Group),<br>Sodium channel protein type 1 subunit alpha,<br>Glutamate receptor ionotropic, kainate 1,<br>Carbonic anhydrase 1, 2, 3, 4,<br>Glutamate receptor 1
11	Iproniazid	C11777	depression	N06AF05	-
12	Sulfaguanidine	-	bacillary dysenteryenteric<br>enteric infections	A07AB03	-
13	Streptomycin	19649	tuberculosis<br>tularemia<br>severe M. avium complex, brucellosis, and enterococcal endocarditis	J04AM01,<br>J01GA01,<br>A07AA54,<br>A07AA04	30S ribosomal protein S12,<br>16S ribosomal RNA,<br>Protein-arginine deiminase type-4
14	Orlistat	3034010	obesity	A08AB01	Pancreatic triacylglycerol lipase,<br>Gastric triacylglycerol lipase,<br>Fatty acid synthase
15	Riboflavin	C00255	ariboflavinosis	A11HA04,<br>S01XA26	Riboflavin kinase,<br>Riboflavin synthase,<br>Flavin reductase (NADPH)
16	Cloperastine	2805		R05DB21	-
17	Antazoline	2200	allergic conjunctivitis.	R06AX05,<br>R01AC04	Histamine H1 receptor
18	Ipratropium bromide	657308	Asthma<br>Rhinorrhea<br>bronchospasm<br>sialorrhea	R03BB01,<br>R01AX03,<br>R03AL01,<br>R03AL02	Muscarinic acetylcholine receptor M1, M2, M3

19	Mefloquine	C07633	malaria	P01BF02,<br>P01BC02	Fe(II)-protoporphyrin IX,<br>Hemoglobin subunit alpha,<br>Adenosine receptor A2a
20	Dienestrol	667476	atrophic vaginitis<br>kraurosis vulvae	G03CB01,<br>G03CC02	Estrogen receptor alpha,<br>Sex hormone-binding globulin
21	Latamoxef	C07231	bacterial infection	J01DD06	Penicillin-binding protein 1A, 1B, 3,<br>D-alanyl-D-alanine carboxypeptidase DacB
22	Piperacillin	C14034	polymicrobial infections	J01CR50,<br>J01CA12	Penicillin-binding protein 1b, 2a, 2b, 3
23	Vinblastine	13342	breast cancer<br>testicular cancer<br>lymphomas<br>neuroblastoma<br>mycosis fungoides<br>histiocytosis<br>Kaposi's sarcoma	L01CA01	Tubulin alpha-1A, beta, delta, gamma-1, epsilon chain<br>Transcription factor AP-1
24	Paclitaxel	C07394	Kaposi's sarcoma<br>cancer of the lung<br>ovarian<br>breast	L01CD01	Tubulin beta-1 chain,<br>Apoptosis regulator Bcl-2,<br>Microtubule-associated protein 2, 4, tau,<br>Nuclear receptor subfamily 1 group I member 2
25	Sulindac	C01531	osteoarthritis<br>rheumatoid arthritis<br>ankylosing<br>spondylitis<br>acute painful shoulder<br>acute gouty arthritis.	M01AB02	Prostaglandin G/H synthase 1, 2,<br>Aldose reductase,<br>Mitogen-activated protein kinase 3,<br>Peroxisome proliferator-activated receptor delta<br>Prostaglandin D2 receptor 2<br>Aldo-keto reductase family 1 member B10
26	Flufenamic acid	C13038	decrease pain and blood loss from menstrual periods.	M01AG03	Prostaglandin G/H synthase 1, 2,<br>Aldo-keto reductase family 1 member C3,<br>Androgen receptor,<br>Peroxisome proliferator-activated receptor alpha, gamma
27	Torasemide	41781	edema associated with congestive heart failure, renal or hepatic diseases<br>Hypertension	C03CA04,<br>G01AE10	Solute carrier family 12 member 1, 2
28	Prestwick857	-	trypanocidal drug<br>pentamidinium(2+)	-	-
29	Triflusal	9458	Stroke<br>myocardial infarction.	B01AC18	Prostaglandin G/H synthase 1,<br>Nuclear factor NF-kappa-B p105 subunit,<br>Nitric oxide synthase, inducible,<br>cAMP and cAMP-inhibited cGMP 3',5'-cyclic phosphodiesteras<br>e 10A
30	Cortisone	C00762	allergic reaction arthritis	H02AB10,<br>S01BA03	-
31	Fludrocortisone	31378	Addison's disease<br>salt-losing androgenital syndrome	S02CA07,<br>S01CA06,<br>H02AA02,<br>S03CA05	Mineralocorticoid receptor,<br>Glucocorticoid receptor
32	Deferoxamine	C06940	aluminum toxicity<br>anemia	V03AC01	Iron,<br>Aluminum,<br>Amyloid beta A4 protein
